# Spatial Control of Epsin-induced Clathrin Assembly by Membrane Curvature*[Fn FN3]^♦^

**DOI:** 10.1074/jbc.M115.653394

**Published:** 2015-04-02

**Authors:** Sachin S. Holkar, Sukrut C. Kamerkar, Thomas J. Pucadyil

**Affiliations:** From the Indian Institute of Science Education and Research, Dr. Homi Bhabha Road, Pashan, Pune 411 008, India

**Keywords:** clathrin, endocytosis, fluorescence, membrane structure, microscopy, epsin, membrane curvature sensing

## Abstract

Epsins belong to the family of highly conserved clathrin-associated sorting proteins that are indispensable for clathrin-mediated endocytosis, but their precise functions remain unclear. We have developed an assay system of budded supported membrane tubes displaying planar and highly curved membrane surfaces to analyze intrinsic membrane curvature preference shown by clathrin-associated sorting proteins. Using real-time fluorescence microscopy, we find that epsin preferentially partitions to and assembles clathrin on highly curved membrane surfaces. Sorting of epsin to regions of high curvature strictly depends on binding to phosphatidylinositol 4,5-bisphosphate. Fluorescently labeled clathrins rapidly assemble as foci, which in turn cluster epsin, while maintaining tube integrity. Clathrin foci grow in intensity with a typical time constant of ∼75 s, similar to the time scales for coated pit formation seen in cells. Epsin therefore effectively senses membrane curvature to spatially control clathrin assembly. Our results highlight the potential role of membrane curvature in orchestrating the myriad molecular interactions necessary for the success of clathrin-mediated membrane budding.

## Teaser

**Background:** The clathrin adaptor epsin is indispensable for clathrin-mediated endocytosis, but the mechanism by which it regulates clathrin assembly remains unclear.

**Results:** Epsin shows a preference to localize to regions of high membrane curvature.

**Conclusion:** Epsin's membrane curvature sensing directs clathrin assembly to highly curved membranes.

**Significance:** Membrane curvature could determine the hierarchy of molecular interactions during clathrin-mediated membrane budding.

## Introduction

**FIGURE 1.**
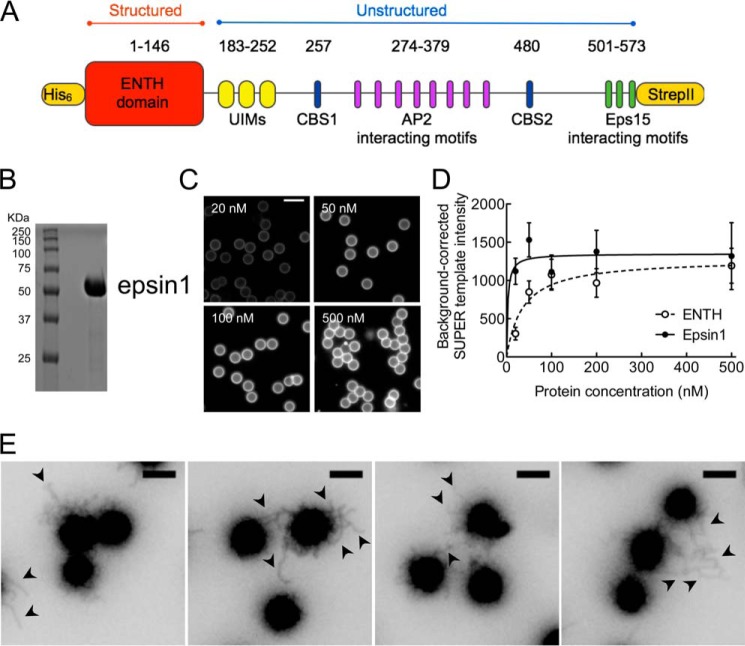
**Membrane binding and tubulation by epsin.** *A*, domain organization of epsin1. *UIMs*, ubiquitin-interacting motifs. *B*, SDS-PAGE-resolved and Coomassie Blue-stained epsin1 protein used in the study. *C*, fluorescence micrographs of SUPER templates incubated with increasing concentrations of Alexa Fluor 488 maleimide-labeled epsin1. *Scale bar* = 10 μm. *D*, background-corrected fluorescence associated with SUPER templates of Alexa Fluor 488 maleimide-labeled epsin1 (*closed circles*) and ENTH domain (*open circles*) fitted to a one-site binding equation. *Error bars* indicate mean ± S.D. *E*, panel showing various fields of tubulated (*black arrowheads*) SUPER templates upon incubation with 200 nm epsin1. Images are inverted in contrast for clarity. *Scale bars* = 5 μm.

Clathrin-mediated endocytosis (CME)^3^ is the major pathway for internalization of membrane proteins from the plasma membrane. CME involves a complex hierarchy of interactions between clathrin, its adaptors, and numerous accessory factors that coordinate and catalyze partial reactions leading to cargo sorting, membrane budding, and fission (1–3). Epsins belong to the family of highly conserved adaptor proteins for ubiquitin-tagged membrane proteins (4, 5) and are indispensable for the turnover of clathrin-coated pits on the plasma membrane (6, 7). Their functions are attributed to a membrane-active N-terminal ENTH domain, which binds phosphatidylinositol 4,5-bisphosphate (PIP_2_) with high affinity (8–11), and an unstructured C-terminal region that contains polyubiquitin-, clathrin-, AP2-, and Eps15-binding sites (see Fig. 1*A*) (4, 12, 13). The precise role of epsins in CME remains unclear. Epsin knockdown/knockout or antibody-mediated depletion results in the accumulation of multiheaded clathrin-coated structures (14) or shallow membrane buds (6, 15), which suggest functions subsequent to nucleation and assembly of clathrin-coated pits. However, minimal biochemical reconstitution assays have demonstrated a capacity for epsin to recruit and assemble clathrin on the membrane leading to sculpting of coated buds (11, 16), implying a pioneering role in CME. All *in vitro* biochemical reconstitution efforts to date have relied on end-point electron microscopic analysis of the clathrin assembly reaction on essentially planar membranes (11, 16–18). A reconstitution scheme that accounts for not just the complexity inherent in interactions among the myriad proteins but also the continuum of membrane curvatures generated during CME could reconcile the apparent disparity in our understanding of epsin function. We tested epsin1 (hereafter referred to as epsin) for its clathrin assembly properties using real-time fluorescence microscopic assays on novel membrane templates that mimic curved membrane intermediates generated during CME.

## Experimental Procedures

### 

#### 

##### Expression, Purification, and Fluorescent Labeling of Proteins

Rat epsin1 and GST-auxilin^547–910^ were kind gifts from Ernst Ungewickell. ENTH domain (2–156), epsin1(L6W), and clathrin-binding site mutants (CBS1: ^257^LMDLAD to ^257^AAAAA and CBS2: ^480^LVDLD to ^480^AAAAA) were generated by site-directed mutagenesis. Bovine HSC70 (Hsc70.RSET.FL.wt(NarI)) was a gift from David McKay (Addgene plasmid 12532 (19)). Except for GST-auxilin^547–910^, all other constructs were cloned as N-terminal His_6_- and C-terminal StrepII-tagged fusions and confirmed by sequencing. All proteins were expressed in BL21(DE3) at 18 °C in autoinduction medium (Formedium, Norfolk, UK). Frozen bacterial pellets were resuspended in HEPES-buffered saline (20 mm HEPES, 150 mm NaCl, pH 7.4), supplemented with a protease inhibitor cocktail (Roche Applied Science), and lysed by sonication. Proteins were first purified on a HisPur cobalt resin (Thermo Scientific) followed by a StrepTrap HP column (GE Healthcare Life Sciences) according to standard procedures. GST-auxilin^547–910^ was purified using glutathione-Sepharose beads (GE Healthcare Life Sciences). Purified proteins were dialyzed overnight against HKS (20 mm HEPES, 150 mm KCl, pH 7.4), supplemented with 10% v/v glycerol, flash-frozen in liquid N_2_, and stored at −80 °C. Clathrin was extracted from clathrin-coated vesicles isolated from goat brains as described earlier (20), with few modifications. Briefly, ∼110 g of brain tissue was cleaned in cold PBS to remove meninges and blood vessels. The tissue was chopped into small pieces and homogenized in an equal volume of assembly buffer (100 mm MES, 1 mm EGTA, 0.5 mm MgCl_2_, pH 6.8) using a Waring blender. The homogenate was spun at 17,700 × *g* for 30 min at 4 °C. The supernatant was isolated and spun at 70,000 × *g* for 1 h at 4 °C. The resultant pellet was suspended in a minimum volume of assembly buffer using a Dounce homogenizer and mixed with an equal volume of 12.5% w/v Ficoll-400 (Sigma) and 12.5% w/v sucrose solution (both prepared in assembly buffer). The suspension was spun at 41,400 × *g* for 40 min at 4 °C, and the resultant supernatant containing clathrin-coated vesicles was diluted 5-fold with assembly buffer supplemented with 0.1 mm PMSF and spun again at 85,195 × *g* for 1 h at 4 °C. The pellet containing clathrin-coated vesicles was suspended in a minimum volume of disassembly buffer (10 mm Tris-Cl pH 8.0, 1 mm DTT, 1 mm PMSF) using a Dounce homogenizer. Samples were left at room temperature for 2–3 h and spun at 100,000 × *g* for 1 h at room temperature. The supernatant was dialyzed overnight against buffer A (25 mm Tris-Cl, 75 mm NaCl, pH 8.0), spun at 100,000 × *g* to remove aggregates, and further purified on a Q Sepharose column (GE Healthcare Life Sciences). Clathrin was eluted with a linear gradient of buffer B (25 mm Tris-Cl, 1 m NaCl, pH 8.0). Peak fractions containing clathrin were pooled, precipitated with 30% (NH_4_)_2_SO_4_ at 4 °C, resuspended in disassembly buffer with 10% v/v glycerol, flash-frozen in liquid N_2_, and stored at −80 °C. Purified epsin and clathrin were labeled with 10-fold molar excess of thiol-reactive Alexa Fluor 488 and Texas Red C2 maleimide dyes (Invitrogen), respectively, for 1 h at room temperature and quenched with DTT. Free dye was removed from epsin preparations using SM2 Bio-Beads (Bio-Rad) and from clathrin preparations by extensive dialysis against disassembly buffer. Labeled clathrin was further purified and enriched for assembly-competent triskelia by dialysis against assembly buffer, pelleting cages at 100,000 × *g*, followed by resuspension in disassembly buffer. All labeled proteins were resolved on a 10% SDS-PAGE and judged to be free of unreacted dye, which typically migrates with the dye front. Of the two residues Cys^96^ and Cys^205^ in epsin1, only the latter present in the unstructured C-terminal tail showed efficient labeling. The ENTH domain (2–196) was labeled to negligible extents and required incorporation of an extrinsic Cys residue at the C terminus.

##### PEGylation of Glass Coverslips

Glass coverslips were passivated by covalent attachment of PEG according to earlier studies (21). Briefly, glass coverslips were cleaned with 3 n NaOH for 5 min and rinsed with water. Clean coverslips were treated with piranha solution (concentrated H_2_SO_4_:30% H_2_O_2_ = 3:2 v/v) for 1 h at room temperature, rinsed with water, and dried on a heat block set at 90 °C. Dried coverslips were silanized with neat (3-glycidyloxypropyl)trimethoxysilane (Sigma) for 5 h under vacuum. Silanized coverslips were rinsed with acetone, air-dried, and placed in a glass beaker containing molten PEG400 (Sigma) or PEG8000 (USB) maintained at 90 °C for 48–60 h. Coverslips were rinsed extensively with water and stored dry in a closed container. Coverslips were sequentially cleaned with 1% SDS, water, methanol, and water in between experiments and could be used 4–5 times without significant loss in surface passivation.

##### Membrane Binding Assays

Small volume chambers were prepared by attaching clipped Eppendorf heads to PEGylated coverslips using a silicone adhesive (Dow Corning). SUPER templates were prepared as described earlier (22) and added to solutions containing increasing concentrations of fluorescently labeled proteins, incubated for 10 min, and imaged.

##### Supported Membrane Tubes (SMrT Templates)

Appropriate volumes of lipid stocks (Avanti Polar Lipids) were aliquoted into glass vials, diluted to a final concentration of 1 mm in chloroform, and stored at −80 °C. Lipid stocks contained a trace amount (0.5 mol%) of the fluorescent DiD (Invitrogen) lipid probe. Stocks were brought to room temperature before use. A small aliquot (∼1–5 nmol of total lipid) was spread on a PEGylated glass coverslip and kept under high vacuum for 5 min to remove all traces of chloroform. A ∼35-μl flow cell (Bioptechs) was assembled by placing a 0.1-mm silicone spacer between the PEGylated coverslip and an indium tin oxide-coated slide. The flow cell was filled with filtered and degassed HKS containing 1% w/v BSA (Sigma) and left undisturbed for 10 min at room temperature. Hydration of the dry lipid causes the formation of large vesicles inside the chamber. Supported membrane tubes are created by extrusion of the large vesicles to narrow membrane tubes by flowing excess HKS buffer containing 1% BSA at high (∼30 mm/s particle velocity inside the chamber) flow rates. SMrT templates were judged ready for experiments when the entire membrane reservoir was extruded into tubes that remained lightly tethered to the surface, even in the absence of external buffer flow.

##### Clathrin Assembly Reactions

SMrT templates were first equilibrated in filtered and degassed assay buffer (HKS, 1% BSA + oxygen scavenger mixture of 0.2 mg/ml glucose oxidase (Sigma, G-2133), 0.035 mg/ml catalase (Sigma, C-40), 4.5 mg/ml glucose, and 1 mm DTT). 200 μl of 200 nm Alexa Fluor 488 maleimide-labeled epsin in assay buffer was introduced and incubated with SMrT templates for 10 min. Excess epsin1 was rinsed with 600 μl of assay buffer. The clathrin assembly reaction was monitored in real time by flowing in 200 μl of 40 nm Texas Red-labeled clathrin, freshly diluted in assay buffer or buffer with 1 mm Mg^2+^ containing HSC70 (1 μm), GST-auxilin^547–910^ (1 μm), and ATP (1 mm). All reactions were carried out at 25 °C.

##### Fluorescence Microscopy

Fluorescence imaging was carried out on an Olympus IX71 inverted microscope through a 100×, 1.4 NA oil-immersion objective. Fluorescent probes were excited with an LED light source (Thorlabs), and fluorescence emission was collected through single-band pass filters (Semrock) with excitation/emission wavelength bandpasses of 482 ± 35 nm/536 ± 40 nm for Alexa Fluor 488, 562 ± 40 nm/624 ± 40 nm for Texas Red, and 628 ± 40 nm/692 ± 40 nm for DiD probes on an Evolve 512 EMCCD camera (Photometrics). Image acquisition was controlled by the MetaMorph software (Molecular Devices).

##### Field Emission Scanning Electron Microscopy

Clathrin- or streptavidin-coated SMrT templates were processed for SEM inside the flow cell by first fixing with 3% w/v glutaraldehyde for 10 min. Tubes were solubilized with 1% SDS and rinsed with excess water. Samples were dehydrated by sequentially passing 10, 20, 40, 60, 80, and 100% ethanol. Fluorescence imaging with fluorescently labeled proteins at each step confirmed no gross changes in protein distribution on membrane tubes during sample preparation. The flow cell was disassembled, and the coverslips were kept under vacuum overnight. Samples were gold-coated using a Q150T turbo-pumped sputter coater (Quorum Technologies) and imaged on an Ultra Plus field emission scanning electron microscope (Zeiss) using a 1.9-kV electron beam and secondary electron detector.

##### Image Analysis

Image analysis of fluorescence micrographs and time-lapse sequences were carried out using Fiji (23), and nonlinear regression analyses were carried out using GraphPad Prism. Dead time of the flow cell was estimated *in situ* for each experiment by calculating the onset of fluorescent clathrin into the microscope field using a plateau followed by one-phase exponential rise function. Frames before the calculated onset were removed from time-lapse sequences. Background-corrected kymographs were generated from lines placed across the entire length of the membrane tube. Pixel intensity *versus* time data for all pixels on a kymograph were exported and fitted to a plateau followed by one-phase exponential rise function. Mean time constants were plotted from fits with an *R*^2^ ≥ 0.8, which sorted out artifacts caused by microscope focus drifts and/or flow-induced lateral movement of foci. Contiguous events were sorted out based on considering the fit with the smallest onset, which reduced oversampling of data caused by clathrin assembly extending across multiple pixels.

## Results

### 

#### 

##### Membrane Binding and Tubulation by Epsin

Because the unstructured C-terminal tail of epsin1 is highly prone to proteolysis under recombinant expression conditions (data not shown), we engineered the rat epsin1 cDNA with N-terminal His_6_ and C-terminal StrepII tags to facilitate purification of full-length epsin1 using a tandem affinity capture protocol (Fig. 1, *A* and *B*). When assaying for PIP_2_ binding that measures association of fluorescently labeled epsin to SUPER templates (22) composed of DOPC:DOPS:DOPIP_2_ (85:15:5 mol %) (Fig. 1*C*), we find that epsin's ENTH domain indeed displays high affinity for PIP_2_ (*K_d_* ∼36 nm, Fig. 1*D*), similar to earlier estimates (8–10). Under similar conditions, full-length epsin showed a 10-fold higher binding affinity (*K_d_* ∼3 nm, *p* = 0.04, global Student's *t* test, Fig. 1*D*). SUPER templates are low-tension supported bilayers with excess reservoir and have earlier been used to analyze membrane remodeling by endocytic proteins (24, 25). The addition of 200 nm epsin caused extensive tubulation of SUPER templates (Fig. 1*E*, *black arrowheads*), consistent with earlier studies (10, 11), but showed no apparent signs of vesiculation.

##### Membrane Curvature Sensitivity of Epsin-induced Clathrin Assembly

**FIGURE 2.**
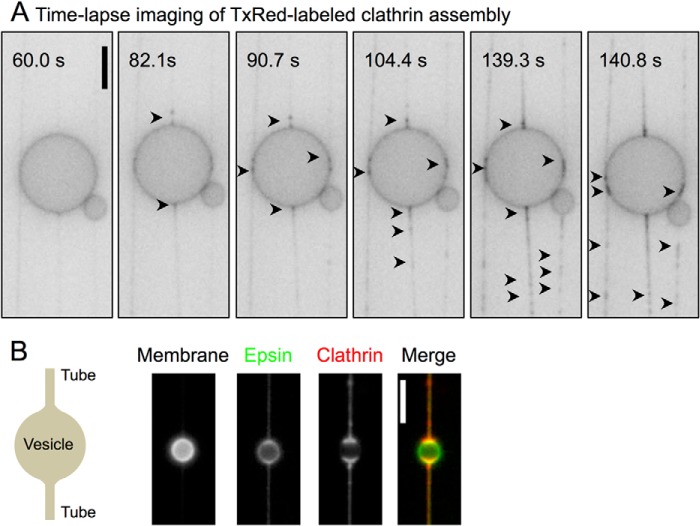
**Real-time visualization of epsin-induced clathrin assembly on membranes.** *A*, panel showing frames from a time-lapse movie of fluorescent clathrin added to epsin-bound vesicles and membrane tubes. Clathrin arrival leads to the formation of foci (*black arrowheads*, see supplemental Movie 1), preferentially on the membrane tubes. Images are inverted in contrast for clarity. *B*, fluorescence micrographs showing the distribution of epsin (*green*) and clathrin (*red*) on coexisting vesicles and membrane tubes. *Scale bars* = 5 μm.

We first recreated epsin-induced clathrin assembly on planar membranes (11, 16) and assayed these reactions using fluorescence microscopy. To minimize osmotic imbalances and mechanical shear imposed on the limiting membrane, vesicles were formed by gentle hydration inside a flow cell. Briefly, a small aliquot (∼1–5 nmol) of DOPC:DOPS:DOPIP_2_:DiD (84.5:15:5:0.5 mol %) lipid mix was dried on a PEGylated glass coverslip (to prevent protein adsorption). Hydration with assay buffer led to the spontaneous formation of large vesicles that were lightly tethered to the surface. During the course of these experiments, we found that buffer flow led to the extrusion of some of the large vesicles into long membrane tubes. Flowing in 200 nm Alexa Fluor 488 maleimide-labeled epsin revealed no significant changes in vesicle morphology (not shown), quite unlike the extensive membrane tubulation seen with low-tension SUPER templates (Fig. 1*E*), suggesting that epsin-induced membrane tubulation is severely impaired on membranes lacking excess membrane reservoir. Unbound epsin was then washed out to reduce possible clathrin cage assembly in solution, and 40 nm Texas Red maleimide-labeled clathrin was introduced into the flow cell. Surprisingly, while monitoring clathrin assembly in real time in fields with coexisting vesicles and tubes previously coated with epsin, we found that although there was a uniform rise in clathrin fluorescence on the vesicle, foci of clathrin started to assemble preferentially on tubes that emanated from and abutted the vesicle (Fig. 2*A*, *black arrowheads*, and supplemental Movie 1). Foci displayed one-dimensional movement, suggesting that they were located on the tubes. Remarkably, foci formation was restricted to tubes, and none appeared on the limiting vesicular membrane during the time course of the assay (Fig. 2*B*). Because tubes represent a membrane surface with high curvature as compared with the limiting vesicular membrane, we probed the curvature sensitivity of epsin-induced clathrin assembly.

##### PIP_2_ Binding-dependent Membrane Curvature Sensing by Epsin Spatially Controls Clathrin Assembly

**FIGURE 3.**
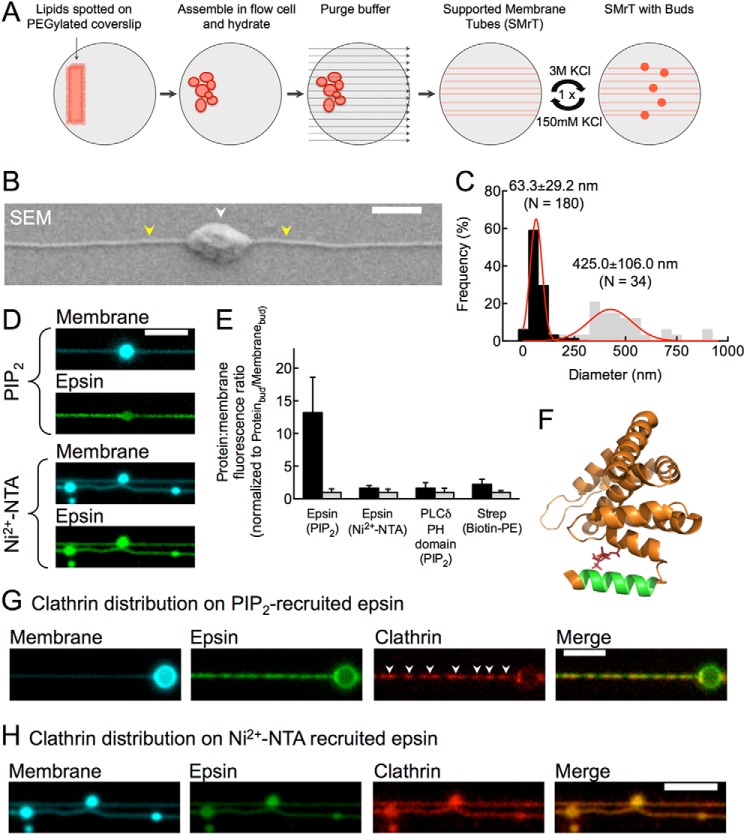
**Epsin-induced clathrin assembly is controlled by membrane curvature.** *A*, schematic of generation of budded SMrT templates. *B*, scanning electron micrograph of streptavidin-bound, fixed, and gold-coated budded SMrT templates. *White* and *yellow arrowheads* mark the bud and tube, respectively. *Scale bar* = 500 nm. *C*, size distribution of tube (*black*) and bud (*gray*) diameters. *D*, fluorescence micrographs of epsin distribution on budded SMrT templates recruited via PIP_2_ or DGS NTA (Ni^2+^). *E*, protein density on membrane tubes (*black*) and buds (*gray*). *Error bars* indicate mean ± S.D. *F*, structure of the epsin1 ENTH domain (Protein Data Bank (PDB) code: 1H0A (11)) with the H0 amphipathic helix shown in *green. G* and *H*, clathrin distribution on membrane tubes coated with epsin recruited via PIP_2_ (*G*) or DGS NTA (Ni^2+^) (*H*). *Scale bars* = 5 μm.

Large vesicles generated as before were extruded by flowing excess buffer at controlled rates to generate an array of membrane tubes lightly resting on a PEGylated glass coverslip (Fig. 3*A*). We refer to these templates as SMrT. Membrane buds were then introduced by exposing SMrT templates to hypertonic shock (see schematic in Fig. 3*A*). Together, these experimental manipulations led to the facile creation of budded SMrT templates that display regions of low (Fig. 3*B*, *white arrowhead*) and high (Fig. 3*B*, *yellow arrowheads*) curvatures, like beads on a string, on a continuous membrane and contained in a flow cell to allow accurate monitoring of reaction kinetics. Scanning electron microscopy of gold-coated and fixed preparations of streptavidin-bound budded SMrT templates composed of DOPC:DOPS:Biotin Cap PE (84:15:5 mol %) indicated tube diameters of 63.3 ± 29.2 nm (mean ± S.D., *n* = 180 profiles on 17 tubes, Fig. 3*C*, *black symbols*) and bud diameters of 425.0 ± 106.0 nm (mean ± S.D., *n* = 34 buds, Fig. 3*C*, *gray symbols*). Accounting for the 4.4- and 4-nm thick streptavidin and gold layers, mean diameters of tubes and buds should be 47 and 408 nm, respectively. Budded SMrT templates therefore represent a novel model membrane system that displays high and low curvatures on a continuous membrane, akin to the topological intermediates generated during CME on the plasma membrane.

We first introduced 200 nm Alexa Fluor 488 maleimide-labeled epsin onto SMrT templates composed of DOPC:DOPS:DOPIP_2_:DiD (84.5:15:5:0.5 mol %). Epsin showed rapid (time constant τ ∼10 s) and uniform binding to tubes (see supplemental Movie 2), which remained intact during the entire course of the assay, in sharp contrast to the previously suggested role of epsins in membrane fission (14). When the same reaction was carried out on budded SMrT templates and imaged after 10 min, epsin fluorescence appeared to be evenly distributed across tubes and buds despite tubes showing weaker lipid (DiD) fluorescence than buds (Fig. 3*D*, PIP_2_ panel). Because the net fluorescence of diffraction-limited, membrane-bound objects is proportional to the underlying membrane surface area (26), we analyzed the epsin:membrane fluorescence ratios to estimate protein densities on tubes and buds. Remarkably, such analysis revealed a 13-fold higher epsin density on tubes as compared with that on buds (ratio_tubes_ = 13.20 ± 5.4, mean ± S.D., *n* = 17; ratio_bud_ = 1.0 ± 0.5, mean ± S.D., *n* = 15, *p* < 0.0001, Student's *t* test, Fig. 3*E*). These results indicate that epsin displays a strong preference to bind membranes with high curvature, and these data are qualitatively consistent with earlier studies with epsin's ENTH domain (27, 28).

PIP_2_ binding coordinates the folding of residues 3–15 in epsin1 into an amphipathic H0 helix (Fig. 2*F*, *green*), which partially inserts into the membrane (8, 10, 11), suggesting a mechanistic basis for epsin's curvature sensitivity (29). Indeed, when recruited via the N-terminal His_6_ tag to SMrT templates composed of 5 mol % DGS NTA (Ni^2+^) lipid instead of PIP_2_ (Fig. 3*D*, Ni^2+^-NTA panel), epsin fluorescence was no longer enriched on tubes and mirrored DiD fluorescence across tubes and buds (ratio_tubes_ = 1.6 ± 0.4, mean ± S.D., *n* = 20; ratio_bud_ = 1.0 ± 0.5, mean ± S.D., *n* = 20, Fig. 3*E*). Thus, epsin's preference for regions of high curvature can be mechanistically attributed to the PIP_2_ binding-dependent folding and membrane insertion of the H0 helix.

Similar analyses carried out with the PIP_2_-binding mEGFP-PLC δ PH domain showed almost equal protein densities on tubes and buds (ratio_tubes_ = 1.6 ± 0.8, mean ± S.D., *n* = 19; ratio_bud_ = 1.0 ± 0.6, mean ± S.D., *n* = 17, Fig. 3*E*), equivalent to that seen with the curvature-insensitive protein streptavidin (30) when recruited via biotinylated lipids (ratio_tubes_ = 2.2 ± 0.8, mean ± S.D., *n* = 20; ratio_bud_ = 1.0 ± 0.5, mean ± S.D., *n* = 20, Fig. 3*E*), suggesting that the enrichment of epsin on tubes was not due to non-uniform PIP_2_ in the membrane. The above described ratiometric analysis reports protein densities at true equilibrium and at concentrations significantly above the binding affinity (∼3 nm, Fig. 1*D*), which together indicate that PIP_2_-recruited epsin has a strong preference to localize to regions with high membrane curvature.

Does epsin's partition preference affect clathrin recruitment on membranes? Remarkably, after incubation for 10 min with epsin-bound budded SMrT templates, clathrin appeared preferentially assembled on tubes and regions connected to the membrane bud (Fig. 3*G*, *white arrowheads*). Importantly, no clathrin foci were apparent on the limiting membrane of the bud, like was seen earlier (Fig. 2*B*). Furthermore, similar assays carried out with DGS NTA (Ni^2+^)-recruited epsin, which renders it insensitive to membrane curvature (Fig. 3*E*), led to uniform distribution of clathrin across tubes and buds (Fig. 3*H*). Thus, the inability of PIP_2_-recruited epsin to assemble clathrin on buds was not due to limiting concentrations of clathrin used in the assay but rather strongly suggests that membrane curvature-dependent sorting of epsin in turn spatially regulates clathrin assembly.

##### Clathrin-binding Site-Clathrin Interactions Are Necessary for Epsin-induced Clathrin Assembly

**FIGURE 4.**
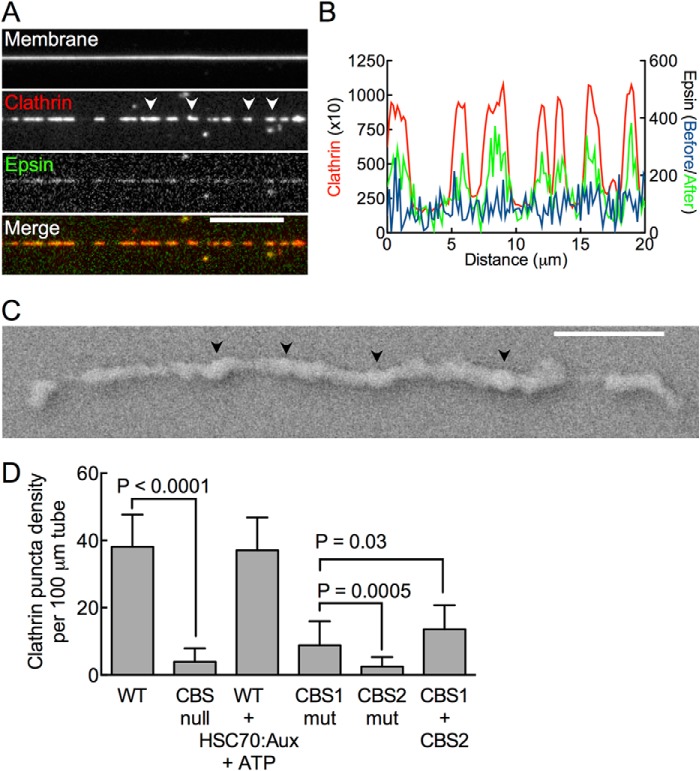
**Specific interactions between the clathrin-binding sites on epsin with clathrin are required for clathrin assembly.** *A*, representative fluorescence micrographs of clathrin and epsin distribution on PIP_2_-containing SMrT templates. *White arrowheads* mark sites of assembled clathrin. *Scale bar* = 10 μm. *B*, fluorescence profiles of epsin distribution on PIP_2_-containing tubes before (*blue*) and after (*green*) a 10-min incubation with clathrin (*red*). *C*, scanning electron micrograph of epsin-induced clathrin assemblies on SMrT templates. *Black arrowheads* mark regions showing pucker in the clathrin coat. *Scale bar* = 400 nm. *D*, clathrin foci density on PIP_2_-containing tubes with epsin and its mutants. *Aux*, auxilin; *mut*, mutant. *Error bars* indicate mean ± S.D.

Clathrin assembly reactions carried out with epsin-bound SMrT templates composed of DOPC:DOPS:DOPIP_2_:DiD (84.5:15:5:0.5 mol %) and imaged after 10 min revealed long polymeric scaffolds (Fig. 4*A*, *white arrowheads*), which strongly coincided with a clustered epsin distribution on the tether (Fig. 4*B*). SEM of scaffolds revealed a continuous protein scaffold with regions showing a puckered appearance (Fig. 4*C*, *black arrowheads*). We were unable to process these reactions for TEM studies as transfer to the EM grids invariably resulted in collapse of scaffolds. The clathrin terminal domain binds epsin at two sites present in the unstructured C-terminal tail: a membrane proximal (CBS1: ^257^LMDLAD) and a distal (CBS2: ^480^LVDLD) site, which have been reported to cooperatively engage in clathrin assembly (31). To test contributions of the clathrin-binding sites to the formation of clathrin scaffolds, we counted numbers of clathrin foci formed on epsin-bound tubes after a 5-min incubation with clathrin because prolonged incubation led to merging of foci (see below). Epsin typically caused robust clathrin assembly on tubes with foci density of ∼40 per 100 μm of tube (Fig. 4*D*, mean ± S.D., *n* = 40 tubes). Importantly, mutating both clathrin-binding sites severely impaired clathrin assembly (CBS null ∼4 per 100 μm of tube, mean ± S.D., *n* = 13 tubes, *p* < 0.0001, Student's *t* test). Carrying out the assembly reaction in the presence of saturating concentrations of the uncoating ATPase HSC70 (1 μm), GST-auxilin^547–910^ (1 μm), and ATP (1 mm), previously shown to disassemble empty clathrin cages (32, 33), had no significant effect on clathrin foci density (WT + HSC70:Aux+ATP = 37 ± 10 per 100 μm of tube, mean ± S.D., *n* = 15 tubes). Mutating the membrane-proximal site (CBS1 mutant) significantly reduced clathrin assembly with foci density dropping by 80% of wild type, whereas mutating the membrane-distal site (CBS2 mutant) reduced clathrin assembly to background levels (Fig. 4*D*). Together, these results indicate that epsin-induced clathrin assembly on SMrT templates is mediated by specific interactions with clathrin-binding sites on epsin, with the membrane distal site being more potent than the membrane proximal site in the assembly process (CBS1 mutant *versus* CBS2 mutant, *p* = 0.0005, Student's *t* test). Coating tubes with an equimolar mix of CBS1 and CBS2 mutants only partially rescued the defect in clathrin assembly seen with CBS1 mutant (CBS1 + CBS2 mutant *versus* CBS1, *p* = 0.03, Student's *t* test), suggesting that the intramolecular placement of the two binding sites is critical for efficient clathrin assembly.

##### Kinetics of Epsin-induced Clathrin Assembly on Tubes

**FIGURE 5.**
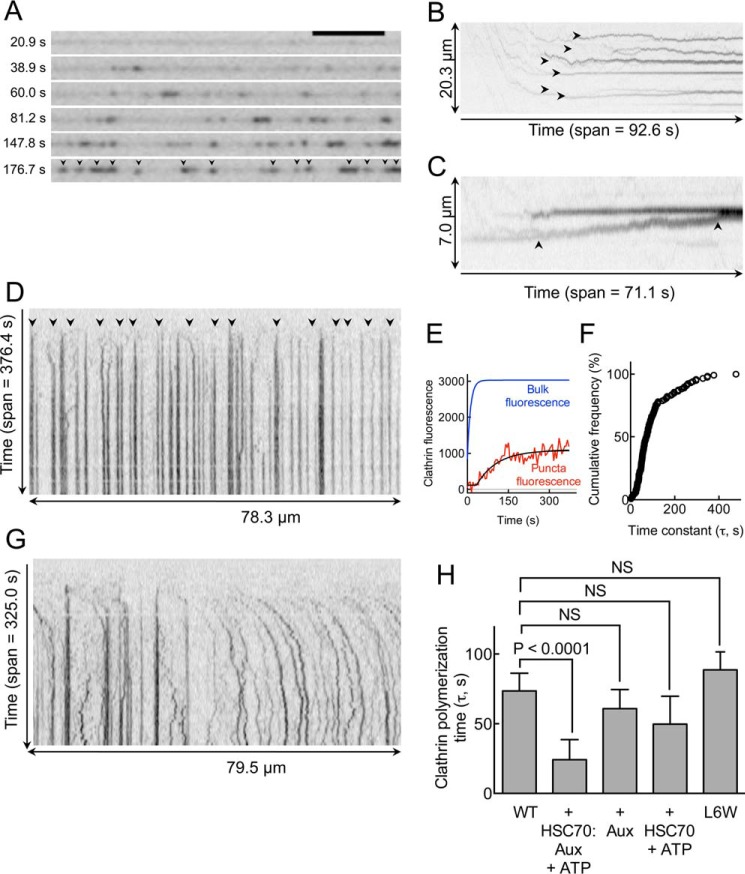
**Kinetics of epsin-induced clathrin assembly.** *A*, frames from a time-lapse movie on epsin-coated tubes showing the formation of fluorescent clathrin foci (*black arrowheads*) (see supplemental Movie 3). *B* and *C*, kymographs generated from clathrin assembly reactions imaged at high temporal resolution (100 ms/frame) showing growth (*black arrowheads* in *B*) and merging (*black arrowheads* in *C*) of clathrin foci. *D*, kymographs from low temporal resolution (5 s/frame) imaging of the entire assembly reaction showing nucleation and growth of numerous clathrin foci (*black arrowheads*). *E*, plot showing single pixel fluorescence traces at a clathrin focus (*red*) and bulk clathrin fluorescence in solution (*blue*) in a typical assembly reaction. *F*, cumulative frequency distribution of time constants for clathrin assembly with epsin. *G*, kymographs from low temporal resolution (5 s/frame) imaging of the entire assembly reaction in the presence of HSC70, GST-auxilin^547–910^, and ATP. All images are inverted in contrast for clarity. *H*, plots comparing the time constants of epsin-induced clathrin assembly in the presence of HSC70, GST-auxilin^547–910^, and ATP and with the L6W mutant of epsin. *Error bars* indicate mean ± S.D. *Aux*, auxilin; *NS*, not significant.

Next, we monitored kinetics of epsin-induced clathrin assembly on SMrT templates at high temporal resolution (100 ms/frame). Arrival of clathrin into the field almost immediately led to the formation of numerous foci (Fig. 5, *A* and *B*, *black arrowheads*, see supplemental Movie 3), indicative of the high degree of cooperativity in clathrin assembly. Aside from the flow-induced directed motion apparent at early time points in the reaction (until about 80 s in supplemental Movie 3), foci were laterally mobile and showed a tendency to merge with each other (Fig. 5*C*, *black arrowheads*) whereupon their mobility was reduced. Imaging at lower temporal resolution (5 s/frame) permitted the generation of kymographs (Fig. 5*D*) of the entire assembly reaction, which were processed (see “Experimental Procedures”) to estimate τ, which reflects the radial (around the tube circumference) assembly kinetics, at single event resolution. A typical trace of clathrin fluorescence increase at a single event is shown in Fig. 5*E*, and it indicates extremely rapid kinetics of assembly that reached saturation in about a minute. Cumulative frequencies of τ showed a long-tailed distribution with a geometric mean of ∼74 s (lower 95% confidence interval = 63 s, upper 95% confidence interval = 86 s, *n* = 118, Fig. 5*F*), similar to the timescales found for the combined nucleation and assembly phases during formation of clathrin-coated pits *in vivo* (34, 35). Importantly, the presence of the uncoating ATPase HSC70, (1 μm) targeted via GST-auxilin^547–910^ (1 μm), appeared to reduce foci merging (see supplemental Movie 4), which resulted in high lateral mobility of foci even at late stages of the assembly reaction, seen in kymographs as wavy traces (Fig. 5*G*). This movement reduced the number of events that could be analyzed for calculating time constants. The few foci that were relatively stable over the entire time course of the assay showed significantly faster kinetics of clathrin assembly with a mean τ of ∼28 s (Fig. 5*H*, *n* = 26, *p* < 0.001, Mann-Whitney *t* test). These events were specific to GST-auxilin^547–910^-mediated recruitment of HSC70 because clathrin polymerization time constants were similar to GST-auxilin^547–910^ or HSC70:ATP alone as was seen with epsin (Fig. 5*H*). Importantly, even in the presence of the epsin (L6W) mutant, which impresses a larger footprint in the membrane (11), formation of clathrin foci proceeded as seen with wild-type epsin with similar assembly kinetics (Fig. 5*G*) and did not disrupt tube integrity (see supplemental Movie 5).

## Discussion

Using time-lapse imaging of clathrin assembly on novel model membrane systems that possibly mimic intermediates with high membrane curvature, such as those found at the dimpled portion or the neck of a nascent coated bud, we provide kinetic analyses of epsin-induced clathrin assembly. Our results reveal a critical role for epsin in sensing and directing rapid and efficient clathrin assembly to regions of high membrane curvature. Once recruited, a likely reason for rapid clathrin assembly could be the high membrane curvature that facilitates assembly of the intrinsically curved clathrin scaffold, as suggested in recent studies where the acquisition of curvature in coated pits is countered by increased membrane tension and bending rigidity (36) or when clathrin assembly reactions are monitored on epsin-coated polystyrene beads (37). Analysis of clathrin assembly on SMrT templates in the presence of HSC70, GST-auxilin^547–910^, and ATP, which disassemble empty clathrin cages (32, 33), provides unanticipated insights into a possible chaperoning role for the uncoating ATPase during adaptor-induced clathrin assembly on membranes. Thus, constant remodeling of the clathrin coat during its assembly in the presence of the uncoating ATPase HSC70 appears to kinetically stimulate clathrin assembly on the membrane, likely by disassembling kinetically trapped intermediates generated during the course of assembly. The reduced tendency for clathrin foci to merge suggests that the disassembly process weakens lateral interactions between coats while allowing foci formation via radial interactions to proceed at high rates. Previous observations (16) of flattening of clathrin-coated membrane buds induced by the uncoating ATPase could likely reflect a scenario where reduced lateral interactions destabilize the coat, leading to collapse of the underlying membrane bud, which is not the case for clathrin assembly on precurved membrane tubes seen here.

The recently proposed role of epsin in fission of endocytic clathrin-coated pits via the membrane-active ENTH domain (14) is not supported in our assays. Membrane tubes, which are structurally closer to the necks of invaginated coated pits, remain intact when uniformly coated with epsin and also in response to clathrin assembly-induced epsin clustering. The pioneering role of epsin in budding of clathrin-coated pits suggested in previous studies likely constitutes a scenario where epsin's intrinsic membrane curvature-sensing abilities are compromised, or only manifest when clustered in the presence of AP2 (11). When provided with a membrane surface displaying high curvature, reactions with epsin alone develop into the formation of small independent clathrin foci at rates that evidently fall within the physiological time scales of coated pit assembly (34, 35). Together, these results strongly suggest that the preferred site for epsin-induced clathrin assembly is a precurved membrane intermediate generated during CME. The shallow membrane curvature generated by the proposed clathrin nucleator module, composed of FCHo, Eps15, and intersectin proteins (38), and further stabilized by AP2, could provide the necessary stimulus to recruit epsin leading to rapid clathrin assembly, a plausible model in light of the suggested enrichment of epsin at the edge of curved clathrin coats (39). Clathrin-coated pits with shallow curvatures are formed even upon significant depletion or complete absence of epsin in cells (6, 14, 15). Nevertheless the coordinated recruitment of endocytic proteins and timely polymerization of actin at endocytic sites appears to be severely compromised (6). Thus, epsin's role could be in coordinating the multiple partial reactions after the acquisition of partial curvature in budded coated pits. We propose that a mechanistic basis for such coordination lies in epsin's ability to sense membrane curvature and direct clathrin assembly during intermediate stages of CME prior to the orchestrated recruitment of accessory factors whose functions together culminate in the successful maturation of a clathrin-coated pit.

## Supplementary Material

Supplemental Data

## Footnotes

3 The abbreviations used are:

CME: clathrin-mediated endocytosis
PIP_2_: phosphatidylinositol 4,5-bisphosphate
CBS: clathrin-binding site
SMrT: supported membrane tubes
ENTH: epsin N-terminal homology
DGS NTA: 1,2-dioleoyl-*sn*-glycero-3-[(*N*-(5-amino-1-carboxypentyl)iminodiacetic acid)succinyl] (nickel salt)
NTA: nitrilotriacetic acid
DOPC: 1,2-dioleoyl-*sn*-glycero-3-phosphocholine
DOPS: 1,2-dioleoyl-*sn*-glycero-3-phospho-l-serine
DOPIP_2_: dioleoyl phosphatidylinositol 4,5-bisphosphate
SUPER: supported bilayers with excess membrane reservoir
DiD: 1,1′-dioctadecyl-3,3,3′,3′-tetramethylindodicarbocyanine perchlorate.
