# Supplementary material for: Spatial Control of Epsin-induced Clathrin Assembly by Membrane Curvature
Source: J Biol Chem. 2015 Apr 2;290(23):14267–76. doi: 10.1074/jbc.M115.653394 (PMC4505496; doi:10.1074/jbc.M115.653394)
Supplement: Supplemental Data [file supp_290_23_14267__index.html]

Spatial control of epsin-induced clathrin assembly by membrane curvature — Spatial Control of Epsin-induced Clathrin Assembly by Membrane Curvature — Membrane Curvature Controls Clathrin Assembly — Supplemental Data 

# Spatial Control of Epsin-induced Clathrin Assembly by Membrane Curvature

## Supplemental Data

**Files in this Data Supplement:**

- Supplementary Movie Legend (.pdf, 50 KB) - Supplementary Movie Legend
- Movie 1 (.mov, 4.0 MB) - Movie showing arrival of fluorescent clathrin and its preferential assembly on tubes.
- Movie 2 (.mov, 2.1 MB) - Movie showing binding of fluorescent epsin to SMrT templates.
- Movie 3 (.mov, 6.8 MB) - Movie showing clathrin foci formation on epsin-coated SMrT templates.
- Movie 4 (.mov, 3.2 MB) - Movie showing clathrin foci formation on epsin-coated SMrT templates in presence of HSC70, GST-auxilin547-910 and ATP.
- Movie 5 (.mov, 3.1 MB) - Movie showing clathrin foci formation on epsin (L6W)-coated SMrT templates.
