# Supplementary material for: Spatial Control of Epsin-induced Clathrin Assembly by Membrane Curvature
Source: J Biol Chem. 2015 Apr 2;290(23):14267–76. doi: 10.1074/jbc.M115.653394 (PMC4505496; doi:10.1074/jbc.M115.653394)
Supplement: Supplemental Data [file supp_M115.653394_jbc.M115.653394-6.pdf]

Spatial control of epsin-induced clathrin assembly by membrane curvature

Sachin S. Holkar, Sukrut C. Kamerkar and Thomas J. Pucadyil

Supplementary Information

Supplementary Movie 1. Movie showing arrival of fluorescent clathrin and its preferential assembly on tubes.

Supplementary Movie 2. Movie showing binding of fluorescent epsin to SMrT templates.

Supplementary Movie 3. Movie showing clathrin foci formation on epsin-coated SMrT templates.

Supplementary Movie 4. Movie showing clathrin foci formation on epsin-coated SMrT templates in presence of HSC70, GST-auxilin$^{547\text{-}910}$ and ATP.

Supplementary Movie 5. Movie showing clathrin foci formation on epsin (L6W)-coated SMrT templates.
